# 4,4′-Bipyridine–cyano­acetic acid (1/2)

**DOI:** 10.1107/S1600536808031322

**Published:** 2008-10-04

**Authors:** Ge Song, Er-Jun Hao, Wei Li

**Affiliations:** a College of Chemistry and Environmental Science, Henan Normal University, Xinxiang 453007, People’s Republic of China

## Abstract

Crystals of the title adduct, C_10_H_8_N_2_·2C_3_H_3_NO_2_, were obtained from a methanol/water solution of cyano­acetic acid and 4,4′-bipyridine at room temperature. In the crystal structure, cyano­acetic acid and centrosymmetric 4,4′-bipyridine mol­ecules are linked by O—H⋯N hydrogen bonds to form three-component supra­molecular adducts. The acidic H atom is almost midway between the O and N atoms of the cyano­acetic acid and bipyridine mol­ecules, with O—H and N—H distances of 1.19 (3) and 1.39 (3) Å, respectively, so that the H-atom transfer is best regarded as partial. The three-component adducts are further inter­connected with neighboring mol­ecules by weak inter­molecular C—H⋯O and C—H⋯N hydrogen bonds and by π–π stacking inter­actions [centroid–centroid distance = 3.7200 (11) Å] to generate a three-dimensional supra­molecular structure.

## Related literature

For similar partial proton transfer from a carbonic acid towards a nitro­gen base, see: Farrell *et al.* (2002*a* ▶,*b* ▶); For C—H⋯O and C—H⋯N hydrogen bonds, see: Balakrishna *et al.* (2005 ▶); Wang *et al.* (2008 ▶).
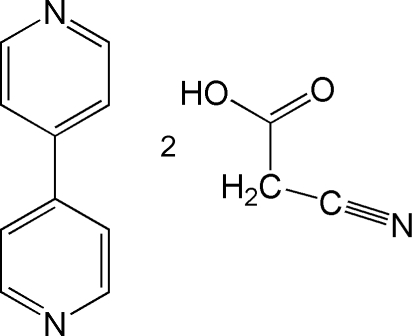


## Experimental

### 

#### Crystal data

C_10_H_8_N_2_·2C_3_H_3_NO_2_
*M* *_r_* = 326.31
Monoclinic, 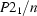

*a* = 4.887 (2) Å
*b* = 21.383 (10) Å
*c* = 7.921 (4) Å
β = 100.664 (8)°
*V* = 813.4 (7) Å^3^
*Z* = 2
Mo *K*α radiation
μ = 0.10 mm^−1^
*T* = 291 (2) K
0.34 × 0.26 × 0.19 mm

#### Data collection

Bruker SMART CCD area-detector diffractometer
Absorption correction: multi-scan (*SADABS*; Bruker, 1997 ▶) *T*_min_ = 0.952, *T*_max_ = 0.982
3537 measured reflections
1487 independent reflections
1153 reflections with *I* > 2σ(*I*)
*R*_int_ = 0.031

#### Refinement

*R*[*F* ^2^ > 2σ(*F* ^2^)] = 0.046
*wR*(*F* ^2^) = 0.133
*S* = 1.04
1487 reflections
112 parameters
H atoms treated by a mixture of independent and constrained refinement
Δρ_max_ = 0.15 e Å^−3^
Δρ_min_ = −0.15 e Å^−3^

### 

Data collection: *SMART* (Bruker, 1997 ▶); cell refinement: *SAINT* (Bruker, 1997 ▶); data reduction: *SAINT*; program(s) used to solve structure: *SHELXS97* (Sheldrick, 2008 ▶); program(s) used to refine structure: *SHELXL97* (Sheldrick, 2008 ▶); molecular graphics: *SHELXTL* (Sheldrick, 2008 ▶); software used to prepare material for publication: *SHELXTL*.

## Supplementary Material

Crystal structure: contains datablocks I, global. DOI: 10.1107/S1600536808031322/zl2142sup1.cif

Structure factors: contains datablocks I. DOI: 10.1107/S1600536808031322/zl2142Isup2.hkl

Additional supplementary materials:  crystallographic information; 3D view; checkCIF report

## Figures and Tables

**Table 1:** Hydrogen-bond geometry (Å, °)

*D*—H⋯*A*	*D*—H	H⋯*A*	*D*⋯*A*	*D*—H⋯*A*
C7—H7*A*⋯N2^i^	0.97	2.92	3.420 (3)	113
C2—H2⋯O2^ii^	0.93	2.62	3.361 (3)	137
C2—H2⋯N2^iii^	0.93	2.75	3.322 (3)	121
O1—H1*D*⋯N1	1.19 (3)	1.39 (3)	2.566 (2)	170 (2)

Symmetry codes: (i) 
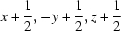
; (ii) 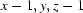; (iii) 
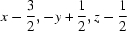
.

**Fig. 1.**
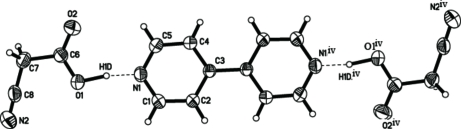
A view of the title compound, showing 30% probability displacement ellipsoids. Symmetry code: (iv) -x, 1 - y,-z.

**Fig. 2.**
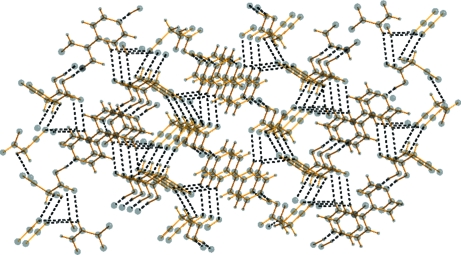
A view of the three-dimensional hydrogen-bonding pattern network.

**Fig. 3.**
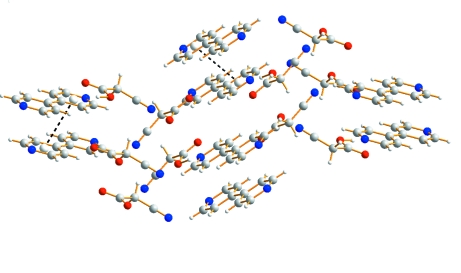
View of the π-π interactions between bipyridine rings in the crystal structure of the title compound.

## supplementary crystallographic information

### Comment

A view of the title structure is shown in Fig. 1. The asymmetric unit consists of one cyanoacetic acid molecule and half a 4,4'-bipyridine molecule. The H1D was found in a Fourier map and its position was refined freely. Within the asymmetric unit, atom H1D is almost mid-way between atoms O1 and N1, so that the H-atom transfer is best regarded as partial. The distances of O1—H1D and N1—H1D are 1.19 (3) Å and 1.39 (3) Å, respectively, which are comparable with literature data (Farrell *et al.*, 2002a,b). Cyanoacetic acid and 4,4'-bipyridine molecules are linked by these O—H···N hydrogen bonds to form 3-component supramolecular adducts.

The 3-compenent adducts interact with neigboring molecules *via* by weak intermolecular C—H···O and C—H···N hydrogen bonds, and by π-π stacking interactions. Within the asymmetric unit, the atoms C2 and C7 act as hydrogen-bond donors, *via* atoms H2, H2, and H7A, to atoms O2^ii^ , N2^iii^ and N2^i^, respectively (symmetry operators: i = *x* + 1/2,-*y* + 1/2,*z* + 1/2; ii = *x* - 1,*y*,*z* -1; iii = *x* - 3/2,-*y* + 1/2,*z* - 1/2). The bond lengths and angles of the above three hydrogen bonds (Table 1) are comparable with literature data (Balakrishna *et al.*, 2005; Wang *et al.*, 2008). These hydrogen bonds, albeit rather weak, link the 3-component supramolecular adducts into a three-dimensional supramolecular structure, which is further stabilized by weak intermolecular π-π stacking interactions, formed by adjacent bipyridine rings (centroid–centroid distance = 3.7200 (11) Å) (Fig. 2 and Fig. 3).

### Experimental

Cyanoacetic acid (0.2 mmol) and 4,4'-bipyridine (0.2 mmol) were dissolved in methanol (5 ml) and water (1 ml) at room temperature. The single crystals of the title compound were obtained from the solution after ten days.

### Refinement

H1D was found in a difference Fourier map and was refined with *U*_iso_(H) = 1.5*U*_eq_(O). All other H atoms were positioned geometrically and treated as riding, with C—H bonding lengths constrained to 0.93 (aromatic CH) or 0.97 Å (methylene CH_2_), and with *U*_iso_(H) = 1.2*U*_eq_(C).

### Crystal data

**Table:**

C_10_H_8_N_2_·2C_3_H_3_NO_2_	*F*(000) = 340
*M**_r_* = 326.31	*D*_x_ = 1.332 Mg m^−^^3^
Monoclinic, *P*2_1_/*n*	Mo *K*α radiation, λ = 0.71073 Å
*a* = 4.887 (2) Å	Cell parameters from 1445 reflections
*b* = 21.383 (10) Å	θ = 2.8–27.1°
*c* = 7.921 (4) Å	µ = 0.10 mm^−^^1^
β = 100.664 (8)°	*T* = 291 K
*V* = 813.4 (7) Å^3^	Block, colorless
*Z* = 2	0.34 × 0.26 × 0.19 mm

### Data collection

**Table:**

Bruker SMART CCD area-detector diffractometer	1487 independent reflections
Radiation source: fine-focus sealed tube	1153 reflections with *I* > 2σ(*I*)
graphite	*R*_int_ = 0.031
φ and ω scans	θ_max_ = 25.5°, θ_min_ = 2.8°
Absorption correction: multi-scan (SADABS; Bruker, 1997)	*h* = −5→5
*T*_min_ = 0.952, *T*_max_ = 0.982	*k* = −25→24
3537 measured reflections	*l* = −9→7

### Refinement

**Table:**

Refinement on *F*^2^	Primary atom site location: structure-invariant direct methods
Least-squares matrix: full	Secondary atom site location: difference Fourier map
*R*[*F*^2^ > 2σ(*F*^2^)] = 0.046	Hydrogen site location: inferred from neighbouring sites
*wR*(*F*^2^) = 0.133	H atoms treated by a mixture of independent and constrained refinement
*S* = 1.04	*w* = 1/[σ^2^(*F*_o_^2^) + (0.0577*P*)^2^ + 0.1963*P*] where *P* = (*F*_o_^2^ + 2*F*_c_^2^)/3
1487 reflections	(Δ/σ)_max_ < 0.001
112 parameters	Δρ_max_ = 0.16 e Å^−^^3^
0 restraints	Δρ_min_ = −0.15 e Å^−^^3^

### Fractional atomic coordinates and isotropic or equivalent isotropic displacement parameters (Å^2^)

**Table:**

	*x*	*y*	*z*	*U*_iso_*/*U*_eq_	
O1	0.9569 (3)	0.33450 (7)	0.35022 (19)	0.0763 (5)	
H1D	0.779 (6)	0.3714 (12)	0.298 (3)	0.114*	
O2	1.0674 (4)	0.40335 (8)	0.5596 (2)	0.1022 (6)	
N1	0.5590 (3)	0.40726 (7)	0.21445 (19)	0.0595 (4)	
N2	1.4242 (5)	0.21975 (10)	0.3693 (3)	0.1017 (7)	
C1	0.3898 (4)	0.38664 (9)	0.0757 (3)	0.0729 (6)	
H1	0.4196	0.3468	0.0354	0.087*	
C2	0.1717 (4)	0.42138 (9)	−0.0118 (3)	0.0682 (6)	
H2	0.0604	0.4052	−0.1100	0.082*	
C3	0.1174 (3)	0.48036 (7)	0.04596 (19)	0.0464 (4)	
C4	0.2939 (4)	0.50109 (9)	0.1930 (2)	0.0652 (5)	
H4	0.2662	0.5402	0.2384	0.078*	
C5	0.5109 (4)	0.46370 (9)	0.2722 (2)	0.0685 (6)	
H5	0.6278	0.4787	0.3700	0.082*	
C6	1.1058 (4)	0.35458 (9)	0.4903 (2)	0.0598 (5)	
C7	1.3428 (4)	0.31156 (10)	0.5682 (3)	0.0707 (6)	
H7A	1.5125	0.3360	0.5955	0.085*	
H7B	1.3038	0.2948	0.6750	0.085*	
C8	1.3896 (4)	0.25990 (10)	0.4580 (3)	0.0699 (6)	

### Atomic displacement parameters (Å^2^)

**Table:**

	*U*^11^	*U*^22^	*U*^33^	*U*^12^	*U*^13^	*U*^23^
O1	0.0708 (9)	0.0714 (9)	0.0772 (9)	0.0193 (7)	−0.0112 (7)	−0.0097 (7)
O2	0.1424 (17)	0.0813 (11)	0.0760 (11)	0.0340 (11)	0.0021 (10)	−0.0164 (8)
N1	0.0558 (9)	0.0595 (9)	0.0611 (9)	0.0096 (7)	0.0053 (7)	0.0059 (7)
N2	0.1006 (16)	0.0809 (13)	0.1175 (17)	0.0300 (12)	0.0043 (13)	−0.0100 (12)
C1	0.0752 (14)	0.0532 (11)	0.0818 (14)	0.0146 (9)	−0.0073 (11)	−0.0079 (9)
C2	0.0700 (13)	0.0540 (10)	0.0708 (12)	0.0087 (9)	−0.0122 (10)	−0.0092 (9)
C3	0.0465 (9)	0.0460 (8)	0.0468 (8)	0.0003 (7)	0.0088 (7)	0.0027 (7)
C4	0.0685 (12)	0.0620 (11)	0.0592 (11)	0.0143 (9)	−0.0038 (9)	−0.0121 (8)
C5	0.0663 (13)	0.0744 (12)	0.0583 (11)	0.0123 (10)	−0.0051 (9)	−0.0077 (9)
C6	0.0676 (12)	0.0595 (10)	0.0536 (10)	0.0058 (9)	0.0143 (9)	0.0026 (8)
C7	0.0672 (13)	0.0827 (13)	0.0586 (11)	0.0084 (10)	0.0025 (9)	0.0020 (9)
C8	0.0613 (12)	0.0669 (12)	0.0773 (14)	0.0145 (10)	0.0021 (10)	0.0099 (10)

### Geometric parameters (Å, °)

**Table:**

O1—C6	1.283 (2)	C3—C4	1.388 (2)
O1—H1D	1.19 (3)	C3—C3^i^	1.498 (3)
O2—C6	1.209 (2)	C4—C5	1.382 (3)
N1—C1	1.323 (2)	C4—H4	0.9300
N1—C5	1.327 (2)	C5—H5	0.9300
N2—C8	1.142 (3)	C6—C7	1.518 (3)
C1—C2	1.377 (3)	C7—C8	1.452 (3)
C1—H1	0.9300	C7—H7A	0.9700
C2—C3	1.384 (2)	C7—H7B	0.9700
C2—H2	0.9300		
			
C6—O1—H1D	109.8 (12)	C5—C4—H4	119.9
C1—N1—C5	117.80 (16)	C3—C4—H4	119.9
C1—N1—H1D	121.6 (10)	N1—C5—C4	122.65 (17)
C5—N1—H1D	120.6 (10)	N1—C5—H5	118.7
C1—N1—H1D	121.6 (10)	C4—C5—H5	118.7
C5—N1—H1D	120.6 (10)	O2—C6—O1	124.78 (19)
N1—C1—C2	123.03 (18)	O2—C6—C7	120.59 (19)
N1—C1—H1	118.5	O1—C6—C7	114.63 (17)
C2—C1—H1	118.5	C8—C7—C6	114.18 (17)
C1—C2—C3	120.22 (18)	C8—C7—H7A	108.7
C1—C2—H2	119.9	C6—C7—H7A	108.7
C3—C2—H2	119.9	C8—C7—H7B	108.7
C2—C3—C4	116.19 (16)	C6—C7—H7B	108.7
C2—C3—C3^i^	121.79 (18)	H7A—C7—H7B	107.6
C4—C3—C3^i^	122.02 (18)	N2—C8—C7	179.0 (2)
C5—C4—C3	120.11 (17)		
			
C5—N1—C1—C2	1.2 (3)	C3^i^—C3—C4—C5	−178.96 (19)
H1D—N1—C1—C2	−179.1 (12)	C1—N1—C5—C4	−0.3 (3)
N1—C1—C2—C3	−1.2 (3)	H1D—N1—C5—C4	−180.0 (12)
C1—C2—C3—C4	0.2 (3)	C3—C4—C5—N1	−0.7 (3)
C1—C2—C3—C3^i^	179.8 (2)	O2—C6—C7—C8	−170.9 (2)
C2—C3—C4—C5	0.7 (3)	O1—C6—C7—C8	9.4 (3)

Symmetry codes: (i) −*x*, −*y*+1, −*z*.

### Hydrogen-bond geometry (Å, °)

**Table:**

*D*—H···*A*	*D*—H	H···*A*	*D*···*A*	*D*—H···*A*
C7—H7A···N2^ii^	0.97	2.92	3.420 (3)	113
C2—H2···O2^iii^	0.93	2.62	3.361 (3)	137
C2—H2···N2^iv^	0.93	2.75	3.322 (3)	121
O1—H1D···N1	1.19 (3)	1.39 (3)	2.566 (2)	170 (2)

Symmetry codes: (ii) *x*+1/2, −*y*+1/2, *z*+1/2; (iii) *x*−1, *y*, *z*−1; (iv) *x*−3/2, −*y*+1/2, *z*−1/2.
